# Cell-free chromatin particles released from dying cancer cells activate immune checkpoints in human lymphocytes: implications for cancer therapy

**DOI:** 10.3389/fimmu.2023.1331491

**Published:** 2024-01-11

**Authors:** Snehal Shabrish, Kavita Pal, Naveen Kumar Khare, Dharana Satsangi, Aishwarya Pilankar, Vishalkumar Jadhav, Sushma Shinde, Nimisha Raphael, Gaurav Sriram, Relestina Lopes, Gorantla V. Raghuram, Harshali Tandel, Indraneel Mittra

**Affiliations:** ^1^ Translational Research Laboratory, Advanced Centre for Treatment, Research and Education in Cancer, Tata Memorial Centre, Mumbai, India; ^2^ Homi Bhabha National Institute, Mumbai, India

**Keywords:** cell death, cellular stress, immunotherapy, cancer, cell-free chromatin deactivators

## Abstract

Immune checkpoint blockade is the exciting breakthrough in cancer, but how immune checkpoints are activated is unknown. We have earlier reported that cell-free chromatin particles (cfChPs) that circulate in blood of cancer patients, or those that are released locally from dying cancer cells, are readily internalized by healthy cells with biological consequences. Here we report that treatment of human lymphocytes with cfChPs isolated from sera of cancer patients led to marked activation of the immune checkpoints PD-1, CTLA-4, LAG-3, NKG2A, and TIM-3. This finding was corroborated *in vivo* in splenocytes of mice when cfChPs were injected intravenously. Significant upregulation of immune checkpoint was also observed when isolated lymphocytes were exposed to conditioned medium containing cfChPs released from hypoxia-induced dying HeLa cells. Immune checkpoint activation could be down-regulated by pre-treating the conditioned media with three different cfChPs deactivating agents. Down-regulation of immune checkpoints by cfChPs deactivating agents may herald a novel form of immunotherapy of cancer.

## Introduction

Immune checkpoint molecules prevent the immune system from indiscriminately attacking self-cells. Immunologically altered cancer cells, on the other hand, can protect themselves from elimination by activating immune checkpoints (1). Consequently, targeting activated immune checkpoints with specific inhibitors is being widely used in the treatment of cancer (2, 3). Several immune checkpoint inhibitors have now been approved by FDA for the treatment of a variety of cancers (4). Although much has been reported on immune checkpoint biology and immune therapy, how immune checkpoints are activated by lymphocytes has not been elucidated.

Several hundred billion to a trillion cells die in the body everyday (5, 6) and the fragmented chromosomal material in the form of cell-free chromatin particles (cfChPs) enter into the extracellular compartment of the body, including into the circulation (7–9). We have earlier reported that cfChPs that circulate in blood of cancer patients, and those that are released locally from dying cancer cells, can readily enter into healthy cells to activate two hallmarks of cancer viz. DNA damage and inflammation (10, 11). Since immune escape by the way of activation of immune checkpoints is another critical hallmark of cancer (12), we investigated whether cfChPs might be the agents that also activate immune checkpoints in human lymphocytes. We approached this question in several ways: 1) by directly treating isolated human T-cells with cfChPs isolated from sera of cancer patients; 2) by intravenously injecting cfChPs isolated from sera of cancer patients into mice; and 3) by using a co-culture system wherein human T cells were exposed to conditioned medium containing cfChPs released from hypoxia-induced dying HeLa cells.

## Materials and methods

### Institutional ethics approval

This study was approved by Institutional Ethics Committee (IEC) of Advanced Centre for Treatment, Research and Education in Cancer (ACTREC), Tata Memorial Centre (TMC) for collection of blood (10mL) from cancer patients and healthy volunteers for isolation of cfChPs and lymphocytes respectively (Approval no. 900520). All participants signed a written informed consent form which was approved by the IEC.

### Animal ethics approval

The experimental protocol of this study was approved by the Institutional Animal Ethics Committee (IAEC) of Advanced Centre for Treatment, Research and Education in Cancer (ACTREC), Tata Memorial Centre (TMC) (Approval no. 12/2020). The experiments were carried out in compliance with the IAEC animal safety guidelines, and with those of ARRIVE guidelines.

ACTREC- IAEC maintains respectful treatment, care and use of animals in scientific research. It aims that the use of animals in research contributes to the advancement of knowledge following the ethical and scientific necessities. All scientists and technicians involved in this study have undergone training in ethical handling and management of animals under supervision of FELASA certified attending veterinarian. Inbred female C57Bl/6 mice were obtained from our Institutional Animal Facility. All mice were maintained in covenant with Institutional Animal Ethics Committee (IAEC) standards. Animals were euthanized at appropriate time points under CO_2_ atmosphere by cervical dislocation under supervision of FELASA trained animal facility personnel.

### Collection of blood samples for cfChPs and lymphocyte isolation

For lymphocyte culture, peripheral blood samples from healthy adult volunteers were collected using Vacutainer TM tubes (Becton-Dickinson Vacutainer Systems, Franklin Lakes, NJ, U.S.A.) containing sodium heparin anticoagulant. For isolation of cfChPs blood was collected from cancer patients in plain vacutainer (VACUETTE blood collection tube Serum Clot Activator PREMIUM).

### Isolation of cfChPs from human sera

cfChPs were isolated from sera of cancer patients according to a protocol described by us earlier (11). In order to maintain inter-experimental consistency, pooled serum (typically from ~5 individuals) was used to isolate cfChPs and were quantified in terms of their DNA content as estimated by the Pico-green quantification assay.

### Fluorescent dual labelling of cfChPs

cfChPs were fluorescently dually labelled in their DNA by Platinum Bright 550 (red) and in their histone H4 with ATTO-TEC 488 (green) according to a protocol described by us earlier (11).

### PBMC isolation

Peripheral blood mononuclear cells (PBMC) were isolated using Ficoll-Hypaque according to standard procedures.

### FACs sorting

PBMCs were stained with FITC-conjugated anti-human CD4 antibody (clone PRA-T4, BD Biosciences Pharmingen, San Jose, CA, USA) and PerCP-conjugated anti-human CD8 antibody (clone SK1, BD Biosciences Pharmingen, San Jose, CA, USA) or with FITC-conjugated anti-human CD3 antibody (clone UCHT1, BD Biosciences Pharmingen, San Jose, CA, USA). Cells were sorted on FACS Aria III (BD Biosciences, San Jose, CA, USA). Data were analyzed using FACS Diva software (version 4.0.1.2; Becton, Dickinson and Company). Sorted cells were seeded in 24-well plates and were allowed to rest overnight at 37°C in humidified atmosphere of 5% CO_2_ prior to stimulation.

### Preparation of cfChPs deactivating agents

The cfChPs deactivating agents used in our study were: 1) anti-histone antibody complexed nanoparticles (CNPs) (13); 2) DNase I from bovine pancreas was procured from Sigma-Aldrich, and 3) a novel pro-oxidant combination of Resveratrol and Copper(R-Cu), which can deactivate/degrade cfChPs via the medium of free-radicals (14–17). The concentrations of R and Cu used in this study were 1mM and 0.0001mM respectively (14).

### Treatment of lymphocytes with cfChPs isolated from sera of cancer patients

Sorted CD4^+^T cells and CD8^+^T cells were plated at a density of 5x10^5^ in 24-well plates containing 1ml of DMEM. After overnight culture, cells were treated with cfChPs (10ng equivalent of DNA) isolated from sera of cancer patients and used for determining upregulation of immune checkpoints.

### Procedure for collecting conditioned media from hypoxia induced dying HeLa cells

A dual chamber system was used to generate conditioned medium containing cfChPs released from dying cells. HeLa cells (~1x10^5^) were seeded on ThinCert^®^ Cell Culture Inserts (pore size 400nm) containing 1.5ml of DMEM and were placed in 6-well culture plate and were incubated overnight at 37°C. The 6-well plate with Thincert^®^ Inserts was transferred to a hypoxia chamber with 1% O_2_ for 48h to induce hypoxic cell death. Sufficient DMEM (~700 μl) was added to the lower chamber of the 6-well plate such that the medium touched the lower surface of the ThinCert^®^ Inserts and plates were placed at 37°C in humidified atmosphere of 5% CO_2_ for 48h under normoxic conditions. This procedure allowed cfChPs <400 nm in size released from the hypoxic HeLa cells to seep into the medium in the lower chamber. Post-incubation, conditioned media from the wells was pooled and was used for further experiments.

### Fluorescent dual labelling of HeLa cells

HeLa cells were dually labeled in their histones (H2B) and DNA. Histone H2B labelling was done for 36h using CellLight^®^ Histone 2BGFP (Thermo Fisher Scientific, MA, USA) and DNA labeling was done for 24h using BrdU (10 μM Sigma Chemicals, MO, USA). The procedure for dual labelling of culture cells has been described by us earlier (10). Dually labeled HeLa cells were seeded on ThinCert^®^ Cell Culture Inserts and cultured in hypoxic conditions (1% O_2_) for 48h. Two hundred and fifty micro-liters of conditioned media containing cfChPs <400nm that had seeped into the lower chamber of the insert was applied to isolated PBMCs in a time course experiment (2h, 4h and 6h). Cells were then washed and processed for fluorescence microscopy to detect presence of fluorescent signals of BrdU and histone H2BGFP in the recipient PBMCs.

### Treatment of cells with conditioned media collected from hypoxic HeLa cells

Conditioned media from hypoxia treated HeLa cells was collected as described above. Sorted T cells were plated at a density of 5x10^5^ in 24-well plate containing 250μL of DMEM media. After overnight culture, cells were treated with 250μL of conditioned media and a time course analysis using qRT-PCR was performed to determine up-regulation of immune checkpoint. In order to confirm that the active agents in the hypoxic media were cfChPs released from the dying HeLa cells, the hypoxic media was pre-treated for 1h with the following cfChPs inhibitors: 1) CNPs (25µg of anti-H4 IgG conjugated nanoparticles) in 125 µL of phosphate buffer; 2) DNase I in PBS to achieve a final concentration of 0.05U/mL; 3) R:Cu in distilled water to achieve a final molar ratio of 1 mM R: 0.0001 mM Cu).

### Analyses of immune checkpoints

#### qRT-PCR

Evaluation of immune checkpoints was performed using qRT-PCR following treatment of human lymphocytes with cfChPs or with media of hypoxic dying HeLa cells as described above. The time points for analyses were: 0h, 30min, 6h, 12h, 24h, 36h and 48h. Appropriate untreated control cells for each time point were analyzed in parallel. Total RNA was isolated using RNeasy Mini Kit (Qiagen, Hilden, Germany) and approximately 1 µg of isolated RNA was converted to cDNA using RT^2^ First Strand Kit (Qiagen, Hilden, Germany). cDNA was diluted (1:10) and used in 10µl reaction volume in duplicates. Real-time PCR was carried out using SYBR Select Master Mix (Applied Biosystems, CA, USA) and all the samples were assayed on a QuantStudioTM 12K Flex Real-Time PCR System (ThermoFisher) using a 384-well block in duplicates. Data were analyzed using a comparative C_T_ method and fold change in mRNA expression was calculated as 2^(-ΔΔC_T_).

#### Immunofluorescence

For detection of immune checkpoints expression, immunofluorescence analysis for five immune checkpoints was performed at peak time points of mRNA expression following treatment of cells. Methodological details of immunofluorescence have been described by us earlier (10). Briefly, slides were mounted with vectashield mounting medium with DAPI (Vector Laboratories) and analyzed on Applied Spectral Imaging system as described by us earlier (10). All experiments were performed in duplicate, and 500 cells were analyzed in each case. Results were expressed as mean ± SEM.

#### Flow cytometry

After 72hrs of cfChPs treatment, PBMCs were labelled with the following antibodies; anti-CD3-FITC (clone:UCHT1), anti-CD-8 PerCP (clone:SK1), anti-PD-1-BV421 (clone:EH12.1), anti-CTLA-4-PE-CF594 (clone:BN13), anti-LAG-3-BUV395 (clone:T47-530), anti-NKG2A-BV421 (clone:131411) and anti-TIM-3-BV786 (clone:7D3). All antibodies were purchased from Becton Dickinson Biosciences, San Jose, CA, USA. Cells were incubated for 20mins in dark at room temperature followed by PBS wash. Samples were acquired on FACSAria III cytometer (Becton Dickinson, USA) and analyzed with *FlowJo*™ *v10.6 Software* (ThreeStar Inc, USA). Lymphocytes were gated on forward and side scatter parameters. At least 20,000 cells were acquired. Untreated T cells reveal a basal expression of immune checkpoints which increases following treatment. Therefore, to determine an increase in percent positive cells, the difference between negative and positive expression was defined by the fluorescence minus one (FMO) method.

### *In vivo* studies

#### Intravenous injection of cfChPs into mice

For the *in vivo* study, 21 mice (3 per group, 7 groups) were injected with cfChPs (100ng dissolved in saline for each mouse) and 3 mice acted as untreated controls. Mice were sacrificed under anesthesia at 6h, 12h, 18h, 24h, 48h, 72h and 96h after cfChPs injection and spleen was removed.

### Preparation of mouse splenocytes

The spleens were minced with a sharp sterile blade, placed in a 40-m nylon cell strainer and pressed with the plunger of a syringe. Splenocytes were suspended in RPMI-1640 supplemented with 5% FBS. Red blood cells were lysed with 1XBD Pharmlyse, washed, and splenocytes were re-suspended in 5% FBS in PBS.

### Analysis of activation of immune checkpoints in splenocytes by flow cytometry

One million splenocytes were stained for 20 min in the dark with the following antibodies: CD3-APC-Cy7 (Clone: 17A2), CD4-FITC (Clone : GK1.5), CD8-APC (Clone:53-6.7), PD-1-BV510 (Clone:29F.1A12), CTLA-4-PE (Clone : UC10-4B9), NKG2A-PE (Clone:16A11), Tim-3-BV421 (Clone : RMT3-23) and LAG-3-PerCP-Cy5.5 (Clone:C9B7W). All antibodies were purchased from BioLegend company (USA). Samples were acquired on BD FACS Aria III (Becton Dickinson, USA) and analyzed with *FlowJo*™ *v10.6 Software* (ThreeStar Inc, USA) as described above.

### Statistical analysis

All data are presented as Mean ± Standard Error of Mean (SEM). Statistical analysis was performed using GraphPad Prism 8 (GraphPad Software, Inc., USA, Version 8.0). Data were compared using Student’s t*-* test (two tailed, unpaired), one-way ANNOVA and Bonferroni’s multiple comparisons test. *p* < 0.05 was taken as the level of significance.

## Results

### cfChPs are readily internalized by human lymphocytes

In the context of our first approach i.e. of treating lymphocytes with cfChPs from cancer patients, we revisited our earlier experiments wherein we had shown that cfChPs are readily internalized by mouse fibroblast cells (11). We confirm using fluorescent microscopy that, like in case of fibroblast cells, treatment of lymphocytes with dually fluorescently labelled cfChPs, in their DNA with Platinum Bright 550 and in their histones with ATTO-TEC-488, results in their rapid uptake within 2h (**Supplementary Figure 1**).

### cfChPs isolated from sera of cancer patients activate immune checkpoints in human lymphocytes

**Figure 1:**
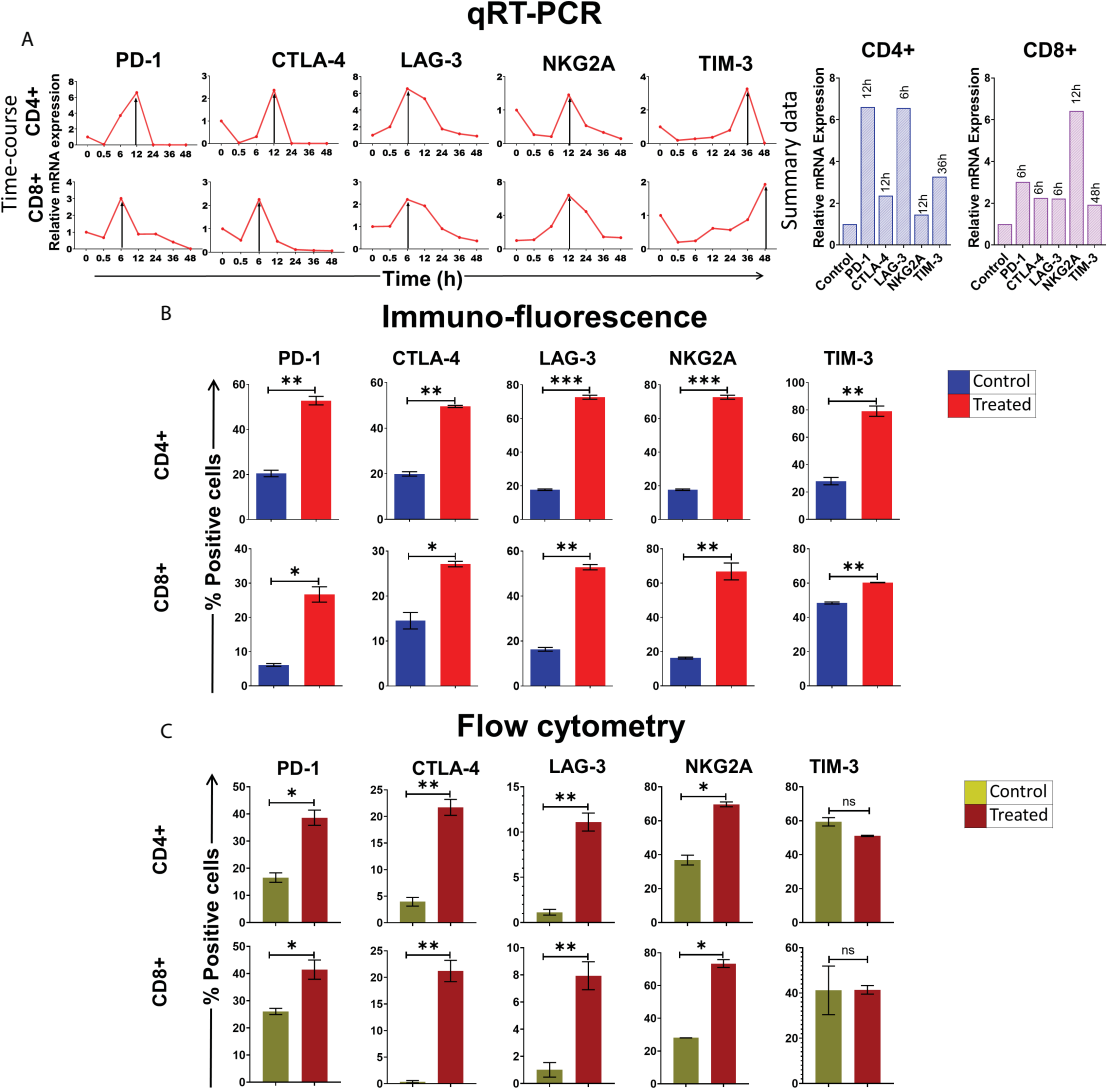
Upregulation of immune checkpoints on human lymphocytes following treatment of cfChPs isolated from sera of cancer patients. **(A)** Line graphs showing time course analysis of mRNA expression of immune checkpoints detected by qRT-PCR in purified CD4+T cells and CD8+T cells treated with cfChPs (10ng). The expression fold change was analyzed using a comparative C_T_ method [2^(-ΔΔC_T_)]. The histograms represent relative mRNA expression at respective peak time points on CD4+T cells and CD8+T cells. **(B)** Histograms depict the results of quantitative Immunofluorescence (IF) analysis of percent positive cells for immune checkpoints on CD4+T and CD8+T-cells at peak time points of mRNA expression. Five hundred cells were examined in duplicate slides and percent biomarker-positive cells were recorded. **(C)** Histograms depict the results of quantitative flow cytometry analysis of surface expression of immune checkpoint on CD4+T and CD8+T-cells at 72hrs post cfChPs treatment. All experiments were performed in duplicates and histograms represent mean ± SEM values. Statistical analyses were performed using a two-tailed student’s unpaired t-test (GraphPad Prism 8). * p<0.05, ** p<0.01, *** p<0.001.

We have earlier reported that cfChPs that circulate in blood of cancer patients, and those that are released locally from dying cancer cells, can readily enter into healthy cells to activate two hallmarks of cancer viz. DNA damage and inflammation (10, 11). Since immune escape by the way of activation of immune checkpoints is another critical hallmark of cancer (12), we investigated whether cfChPs might be the agents that also activate immune checkpoints in human lymphocytes. To investigate this possibility, we treated sorted CD4^+^T and CD8^+^T cells with cfChPs (10ng) from cancer patients and performed a time course analyses using qRT-PCR to detect 5 immune checkpoints. All five immune checkpoints, viz. PD-1, CTLA-4, LAG-3, NKG2A and TIM-3, were found to be markedly up-regulated, albeit at different time points (**Figure 1A**). We validated the above finding of immune checkpoint expression by two methods: 1) by immune-florescence at peak time points of mRNA expression following cfChPs treatment, and 2) by flow cytometry at 72 h. (**Figures 1B, C**; **Supplementary Figures 2A, B, D, E** **;** **Supplementary Table 1**).

### cfChPs activate immune checkpoints as a stress response by lymphocytes

**Figure 2:**
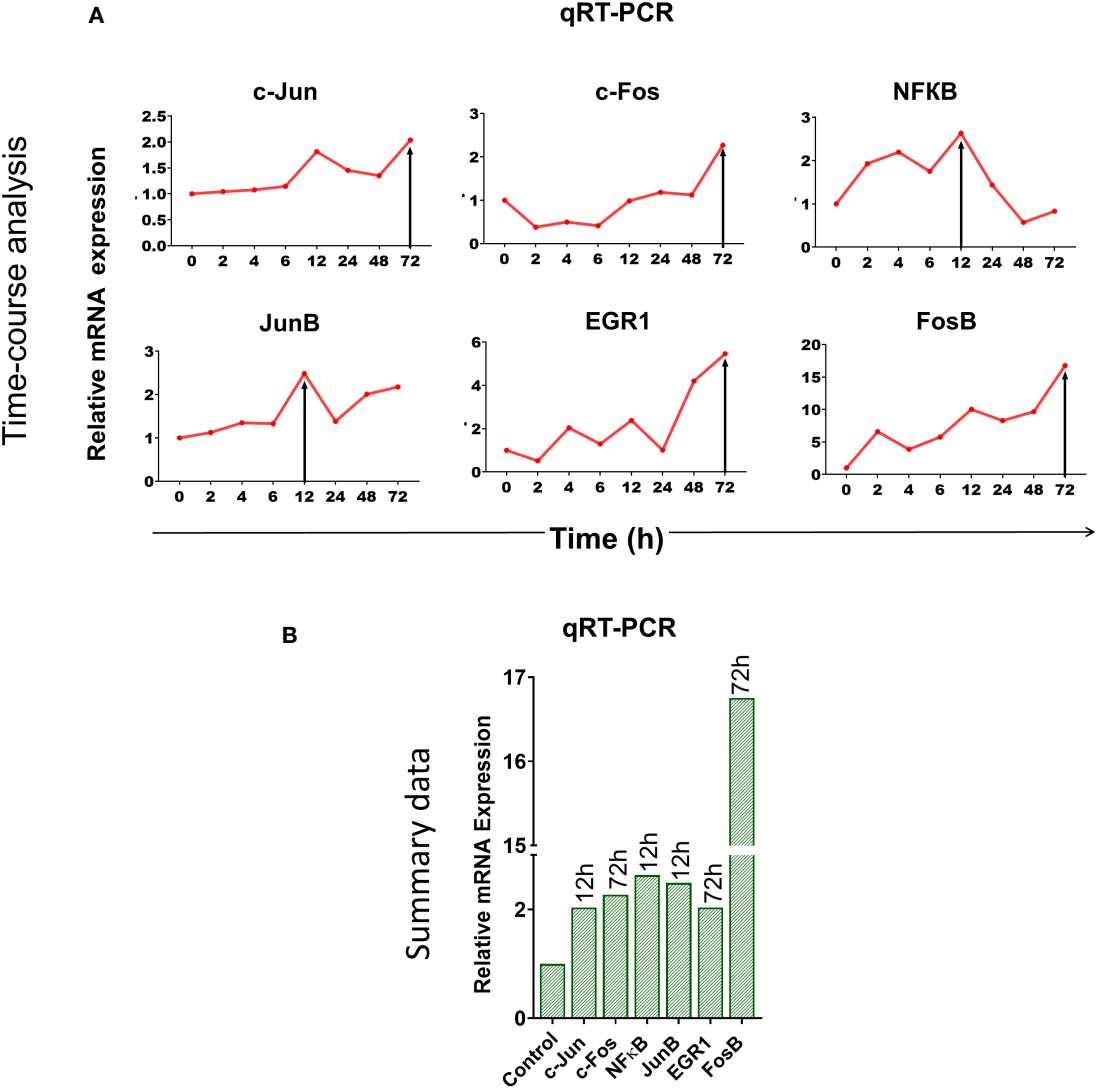
Upregulation of stress-related markers in human PBMCs following treatment of cfChPs isolated from sera of cancer patients. mRNA expression of six stress-related markers in PBMCs was detected by qRT-PCR and analyzed using a comparative C_T_ method. **(A)** Line graphs represent results of time-course analyses calculated as expression fold [2^(-ΔΔC_T_)]. **(B)** Histograms represent relative mRNA expression of stress-related markers at respective peak time points.

We wondered whether immune checkpoint activation might be a stress response of the cell to DNA damage inflicted by cfChPs (10, 11). To this end, we examined six different stress markers that are involved in transcriptional regulation of immune checkpoints (18–20) viz. c-Jun, c-Fos, NFКB, JunB, EGR-1 and FosB by qRT-PCR after treating PBMCs with cfChPs (10ng) isolated from sera of cancer patients. A time-course analysis revealed marked activation of all six stress markers, albeit at different time points (**Figure 2**). These data provided suggestive evidence that cfChPs that circulate in blood of cancer patients activate immune checkpoints in human T cells ostensibly as a response to cellular stress.

### cfChPs upregulate immune checkpoints *in vivo*

**Figure 3:**
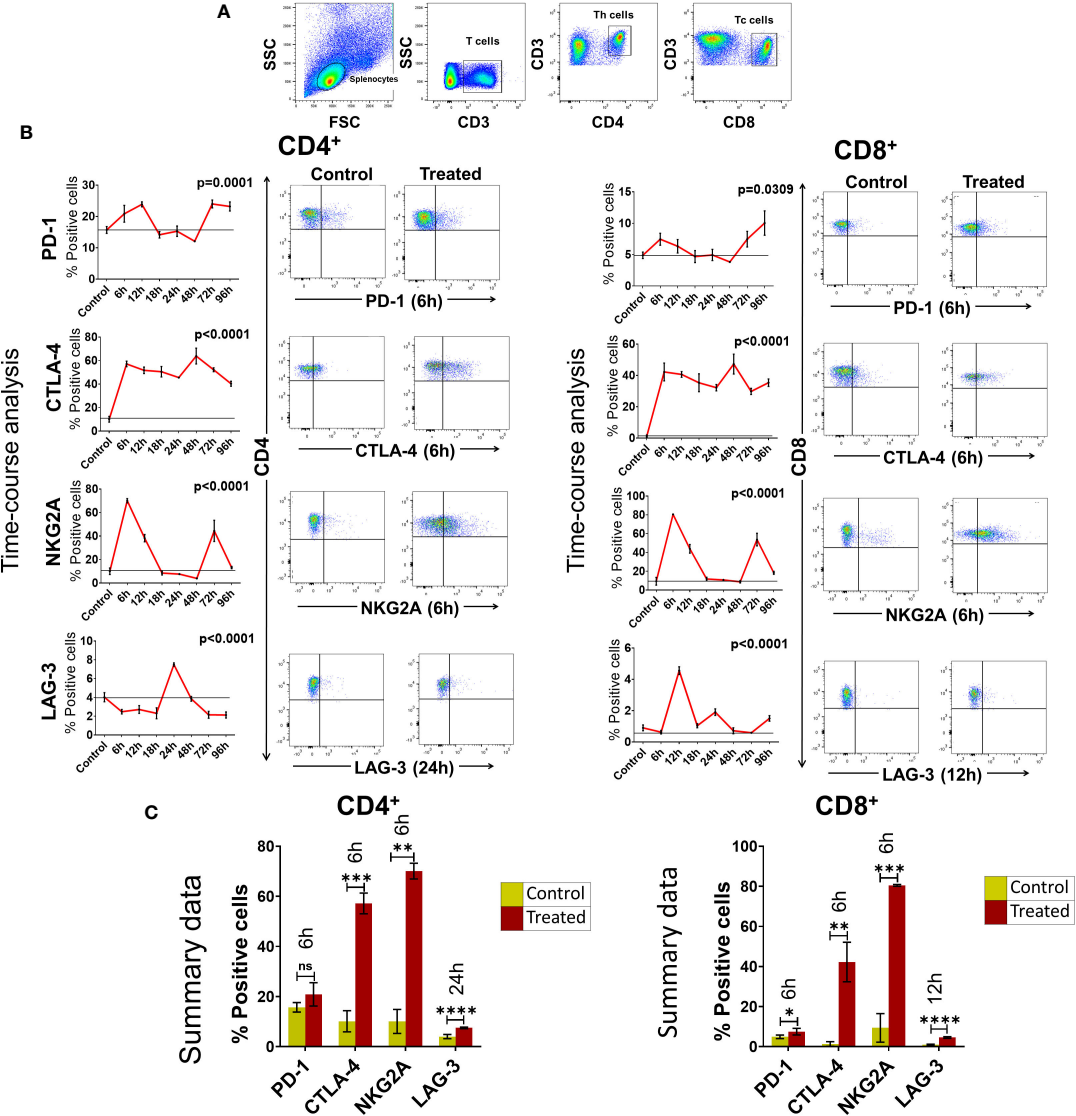
Upregulation of immune checkpoints on splenic lymphocytes of mice following intravenous injection of cfChPs (100ng) isolated from sera of cancer patients. **(A)** Gating of splenic lymphocytes on forward/side scatter, T-cells were identified by using CD3 antibodies, Th cells were identified by using CD4 antibodies and Tc cells were identified by using CD8 antibodies. **(B)** Line graphs showing results of time course analysis of surface expression of various immune checkpoints on CD4^+^T cells and CD8^+^T cells by flow cytometry (N=3 at each time point) (left-hand panels). Representative flow cytometry plots at respective peak time points are given in right- hand panels. Percent expression of each immune checkpoint was compared with untreated controls. **(C)** Histograms represent immune checkpoint expression on CD4+T cells and CD8+T cells at respective peak time points determined by flow cytometry in control and treated mouse splenocytes. Statistical analyses were performed using Bonferroni’s multiple comparisons test (GraphPad Version 8) to compare immune checkpoint expression on T-cells at different time-points in response to cfChPs. A two-tailed unpaired student’s *t*-test was used to compare immune checkpoint expression on T-cells of untreated and treated mice at peak time points of expression. * p<0.05, ** p<0.01, *** p<0.001, **** p<0.0001.

We next examined if cfChPs could activate immune checkpoints *in vivo.* Intravenous injection into mice of cfChPs (100ng) isolated from sera of cancer patients led to marked activation of several immune checkpoints in their splenocytes. A time course analysis by flow cytometry of isolated splenocytes detected significant increased surface expression of four out of five immune checkpoints on CD4^+^T and CD8^+^T cells. Time points at which immune checkpoints were activated were variable, viz. PD-1 at 6h, CTLA-4 at 6h, NKG2A at 6h and LAG-3 at 24h. Expression of TIM-3 was not detected on T cells in experiments lasting 96h (**Figure 3**). The variability in time-points of immune checkpoint activation on mouse splenocytes could be attributed to their distinct expression on different cell populations. For example, PD-1 is largely expressed on activated T and B cells and on monocytes; CTLA-4 is expressed on activated T-cells; LAG-3 is expressed on activated T, B, NK, DCs and monocytes; NKG2A is expressed on CD8+T and NK cells, while TIM-3 is expressed on monocytes, NK cells, Th1, Tc1 and Treg cells (21, 22).

### cfChPs released from dying HeLa cells are readily internalized by human lymphocytes

**Figure 4:**
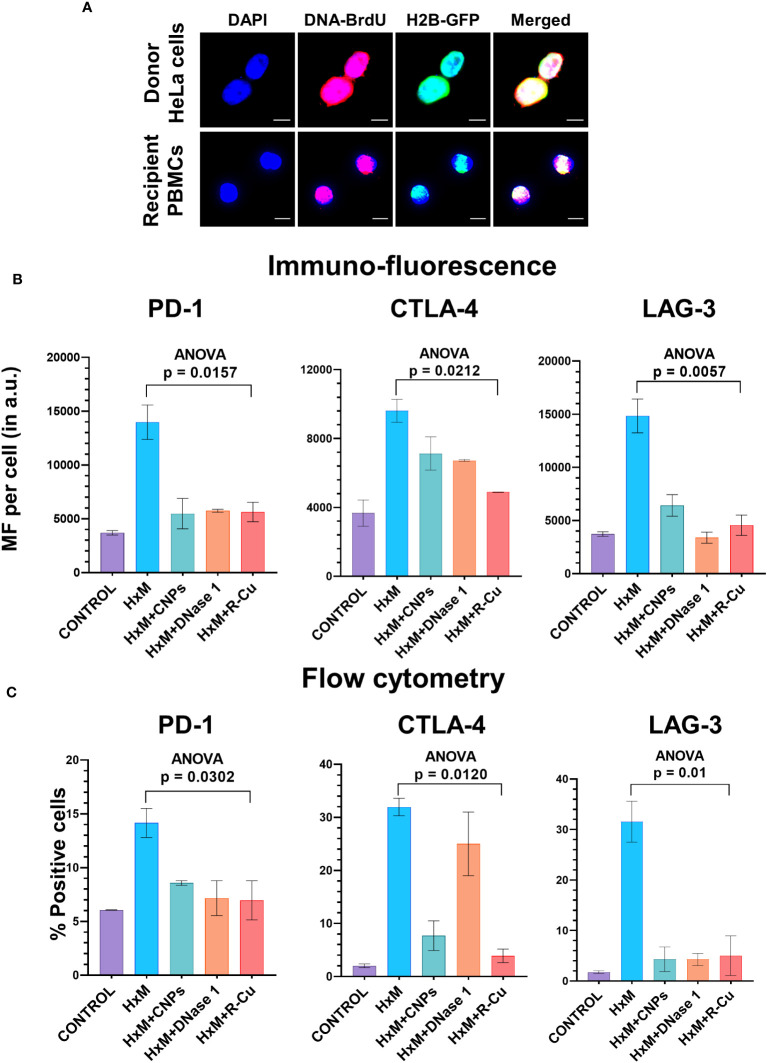
Upregulation of immune checkpoints by cfChPs released from hypoxia- induced dying HeLa cells and their abrogation by cfChPs deactivating agents. **(A)** Uptake by PBMCs of fluorescent cfChPs released from fluorescently dually labelled dying HeLa cells in their DNA with BrdU and in their histones with CellLight^®^ Histone 2BGFP. A dual chamber system of Thincert®Cell Inserts was used and cfChPs released from hypoxia-induced dying HeLa cells were collected from the lower chamber (200uL) and added to PBMCs as described in the methods section. Fluorescence microscopy at 4h detected copious presence of dually labelled cfChPs, released from dying HeLa cells, within the nuclei of PBMCs; **(B)** Results of quantitative IF analysis of upregulation of three immune checkpoints at peak time points of mRNA expression (**Figure 3A**) and their inhibition by concurrent treatment with the cfChPs deactivators, viz. CNPs, DNase I and R-Cu; **(C)** Results of quantitative flow cytometry analysis of upregulation of three immune checkpoints at peak time points of mRNA expression (**Figure 3A**) and their inhibition by concurrent treatment with the cfChPs deactivators, viz. CNPs, DNase I and R-Cu. Experiments were performed in duplicates. Results are represented as mean ± SEM values and data were analyzed using one-way ANOVA (GraphPad Prism 8).

To confirm the above findings in another experimental setting, we devised a method in which isolated T-cells were exposed to conditioned medium containing cfChPs released from hypoxia-induced dying HeLa cells. Such an experimental setting provides further confirmation of the effect of cfChPs on immune checkpoint activation in a more physiological situation as it utilizes cfChPs that has been naturally released into the culture medium from hypoxia induced dying HeLa cells. As a first step, we confirmed that cfChPs released from dying cells are internalized by T-cells. For this, we fluorescently dually labelled HeLa cells in their DNA with BrdU and in their histones with CellLight^®^ Histone 2BGFP (please see Methods) and induced them to undergo apoptosis in a hypoxia chamber for 48h. The culture medium containing dually labelled cfChPs released from the dying HeLa cells was passed through a porous membrane (pore size ~400nm). Isolated human T-cells were incubated in the filtered conditioned medium for 4h (please see Methods). Fluorescence microscopy at 4h detected copious presence of dually labelled cfChPs within PBMCs which had accumulated in their nuclei (**Figure 4A**).

### cfChPs released from dying HeLa cells activate immune checkpoints

Having confirmed that cfChPs released from dying HeLa cells are readily internalized by T-cells, we performed our next experiments using conditioned medium of hypoxia-induced dying but unlabeled HeLa cells. A time course analysis using qRT-PCR revealed marked up-regulation of all five immune checkpoints and six stress markers described above (**Supplementary Figure 3**).

### Activation of immune checkpoint is abrogated by cfChPs deactivating agents

To confirm that cfChPs in the conditioned media were indeed the immune checkpoint activating agents, we pre-treated the conditioned media with three different cfChPs inactivating agents. These included anti-histone antibody complexed nanoparticles (CNPs) which inactivate cfChPs by binding to histones (13); DNase I, which inactivates cfChPs by degrading its DNA component; and a newly described pro-oxidant combination of the nutraceuticals Resveratrol and metallic Copper (R-Cu) which degrades cfChPs through the medium of free radicals (14, 15, 23–25). Immunofluorescence analysis were performed at the peak time point of mRNA expression as defined by qRT-PCR and flow cytometry was performed at 72h after treatment (**Supplementary Figure 3**). We found a highly significant reduction in expression of all three immune checkpoints examined, namely, PD-1, CTLA-4 and LAG-3 (**Figures 4B, C**; **Supplementary Figure 4**). We specifically focused on these three immune checkpoints because inhibitors against these have been approved for clinical use.

## Discussion

Cancer immunotherapy is considered to be a new breakthrough in cancer treatment (4). However, how immune checkpoints are regulated has not been elucidated. We have shown here that cfChPs that circulate in the blood of cancer patients, or those that are released naturally from dying cancer cells, are readily internalized by human lymphocytes leading them to activate immune checkpoints. Cell death has been long associated with immune modulation (26, 27), however, the underlying mechanism(s) remains unclear. Our results suggest that cfChPs that emerge from dying cells to enter into the circulation, or those that are released locally from the dying cells, is the missing link between cell death and immune response.

We wondered whether stress markers that are involved in transcriptional regulation of immune checkpoints (18–20) might be involved in the cfChPs induced activation of immune checkpoints. To this end we show here cfChPs treatment of lymphocytes leads to simultaneous activation of multiple stress markers namely, c-Jun, c-Fos, NFКB, JunB, EGR-1 and FosB.

Abrogation of immune checkpoint activation by the three cfChPs deactivating agents, provided further evidence for the involvement of cfChPs in immune checkpoint activation and suggesting therapeutic possibilities. If confirmed *in vivo*, prevention of immune checkpoints activation may herald the prospect of a novel form of immunotherapy which simultaneously downregulates multiple immune checkpoints all at once. Above all, cfChPs deactivating agents are likely to be far less toxic and less expensive than the immunotherapeutic agents that are currently in use.

Several immune checkpoint inhibitors are currently approved for clinical use. These inhibitors specifically target PD-1 (Pembrolizumab, Nivolumab and Cemiplimab), CTLA-4 (Ipilimumab and tremelimumab) and LAG-3 (Relatlimab) (28). However, each of these agents is specific for a single immune checkpoint, and all of them are associated with considerable toxicity. Their high cost limits their use in countries with limited resources. The three cfChPs deactivating agents used in our study hold the promise of being alternatives to the immune checkpoint inhibitors, especially because of their ability to down-regulate multiple immune checkpoints simultaneously. Of the three agents, CNPs and DNase I, being proteins, are likely to be therapeutically less attractive. On the other hand, a combination of Resveratrol and copper (R-Cu), being commonly used nutraceuticals, would be more attractive, especially since it has already been shown to be effective in multiple therapeutic indications in humans (23–25). For example, the administration of R-Cu to patients with advanced oral cancer down-regulated five immune check-points in the tumor infiltrating lymphocytes (25), immediately suggesting its therapeutic potential in other human cancers. In addition, R-Cu treatment significantly down-regulated nine additional hall-marks of cancer that have been defined by Hanahan and Weinberg (25, 29). R-Cu would have the added advantage of reducing toxic side effects in case it is used as an adjunct to chemotherapy (23, 24). Taken together, these findings make R-Cu a worthy alternative to immune checkpoint inhibitors with which it should be compared in well-designed randomized clinical trials for the treatment of cancer.

## Data availability statement

The original contributions presented in the study are included in the article/**Supplementary Material**. Further inquiries can be directed to the corresponding author.

## Ethics statement

The studies involving humans were approved by Institutional Ethics Committee (IEC) of Advanced Centre for Treatment, Research and Education in Cancer (ACTREC), Tata Memorial Centre (TMC). The studies were conducted in accordance with the local legislation and institutional requirements. The participants provided their written informed consent to participate in this study. The animal study was approved by Institutional Animal Ethics Committee (IAEC) of Advanced Centre for Treatment, Research and Education in Cancer (ACTREC), Tata Memorial Centre (TMC) (Approval no. 12/2020). The study was conducted in accordance with the local legislation and institutional requirements.

## Author contributions

SSha: Conceptualization, Data curation, Formal analysis, Investigation, Methodology, Writing – original draft, Writing – review & editing. KP: Data curation, Methodology, Writing – review & editing. NK: Methodology, Writing – review & editing. DS: Methodology, Writing – review & editing. AP: Methodology, Writing – review & editing. VJ: Methodology, Writing – review & editing. SShi: Methodology, Writing – review & editing. NR: Methodology, Writing – review & editing. GS: Methodology, Writing – review & editing. RL: Methodology, Writing – review & editing. GR: Data curation, Methodology, Supervision, Writing – review & editing. HT: Methodology, Writing – review & editing. IM: Conceptualization, Funding acquisition, Project administration, Supervision, Writing – original draft, Writing – review & editing.

## Supplementary material

Supplementary Figure 1 Rapid internalization by human PBMCs of cfChPs isolated from sera of cancer patients. cfChPs were dually labelled in their DNA and histones with Platinum Bright 550 (red) and ATTO-TEC-488 (green), respectively. Fluorescently dually labeled cfChPs (10ng) when added to PBMCs, fluorescent microscopy detected accumulation of dual labelled cfChPs in their nuclei at 2h.

Supplementary Figure 2 Representative IF images showing upregulation of five immune checkpoints at peak time points of mRNA expression in **(A)** CD4^+^T cells and **(B)** CD8^+^T cells following treatment with cfChPs isolated from sera of cancer patients. Scale bars = 20µm; **(C)** Representative flow cytometry plots of PBMCs showing the gating strategy; **(D)** upregulation of surface expression of immune checkpoint expressions on CD4^+^T cells following 72hrs treatment with cfChPs isolated from sera of cancer patients and; **(E)** upregulation of surface expression of immune checkpoint expressions on CD8^+^T cells following 72hrs treatment with cfChPs isolated from sera of cancer patients.

Supplementary Figure 3 Time-course analysis using qRT-PCR to determine the point of maximum expression of immune checkpoints and stress markers following treatment of PBMCs with conditioned media containing cfChPs released from hypoxia- induced dying HeLa cells. **(A)** Upregulation of immune checkpoints and **(B)** upregulation stress related markers.

Supplementary Figure 4 Abrogation of immune checkpoints by cfChPs deactivators **(A)** Representative IF images showing immune checkpoint expression in T cells following treatment of PBMCs with conditioned media containing cfChPs released from hypoxia-induced dying HeLa cells. Pre-treatment of conditioned medium with CNPs (25μg), DNase I (0.005U) and R-Cu (1:10^-4^) significantly reduced the expression of immune checkpoints. **(B)** Representative flow cytometry plots showing immune checkpoint expression in T cells following treatment of PBMCs with conditioned media containing cfChPs released from hypoxia-induced dying HeLa cells. Pre-treatment of conditioned medium with CNPs (25μg), DNase I (0.005U) and R-Cu (1:10^-4^) significantly reduced the expression of immune checkpoints.
