# Supplementary figures and images for: Cell-free chromatin particles released from dying cancer cells activate immune checkpoints in human lymphocytes: implications for cancer therapy
Source: Front Immunol. 2024 Jan 11;14:1331491. doi: 10.3389/fimmu.2023.1331491 (PMC10808321; doi:10.3389/fimmu.2023.1331491)

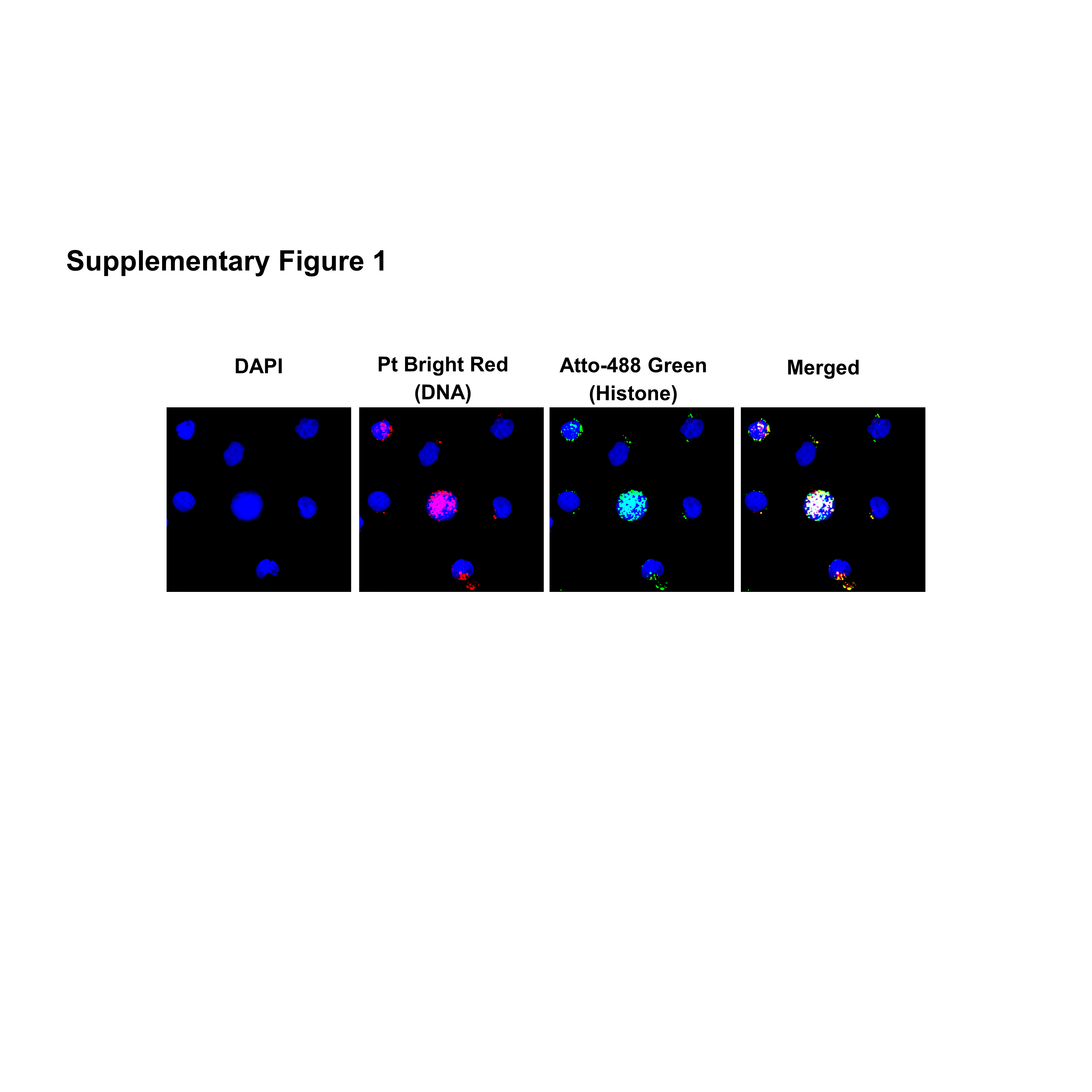

Supplement: Supplementary Figure 1 — Rapid internalization by human PBMCs of cfChPs isolated from sera of cancer patients. cfChPs were dually labelled in their DNA and histones with Platinum Bright 550 (red) and ATTO-TEC-488 (green), respectively. Fluorescently dually labeled cfChPs (10ng) when added to PBMCs, fluorescent microscopy detected accumulation of dual labelled cfChPs in their nuclei at 2h. [file Image_1.tif]

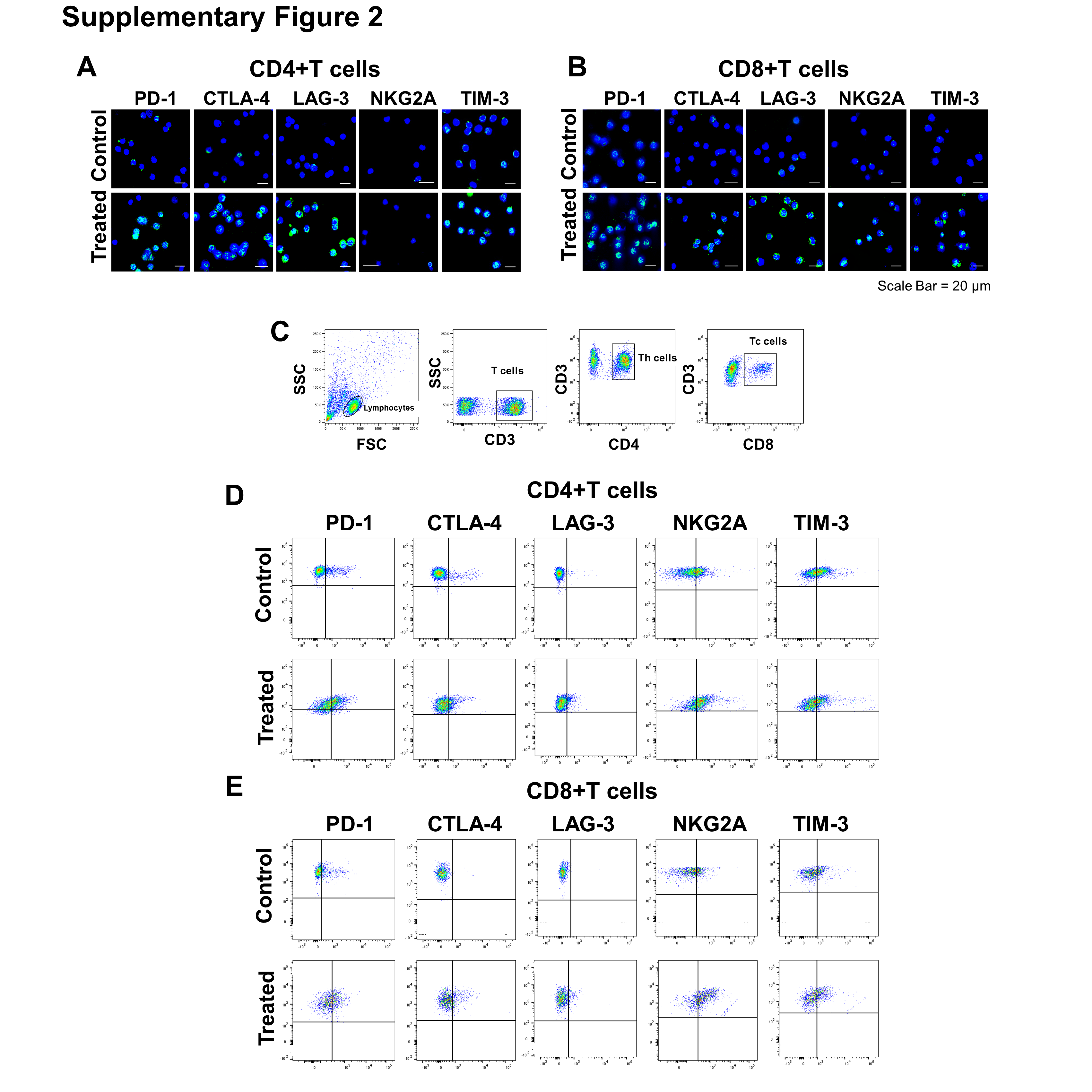

Supplement: Supplementary Figure 2 — Representative IF images showing upregulation of five immune checkpoints at peak time points of mRNA expression in (A) CD4+T cells and (B) CD8+T cells following treatment with cfChPs isolated from sera of cancer patients. Scale bars = 20µm; (C) Representative flow cytometry plots of PBMCs showing the gating strategy; (D) upregulation of surface expression of immune checkpoint expressions on CD4+T cells following 72hrs treatment with cfChPs isolated from sera of cancer patients and; (E) upregulation of surface expression of immune checkpoint expressions on CD8+T cells following 72hrs treatment with cfChPs isolated from sera of cancer patients. [file Image_2.tif]

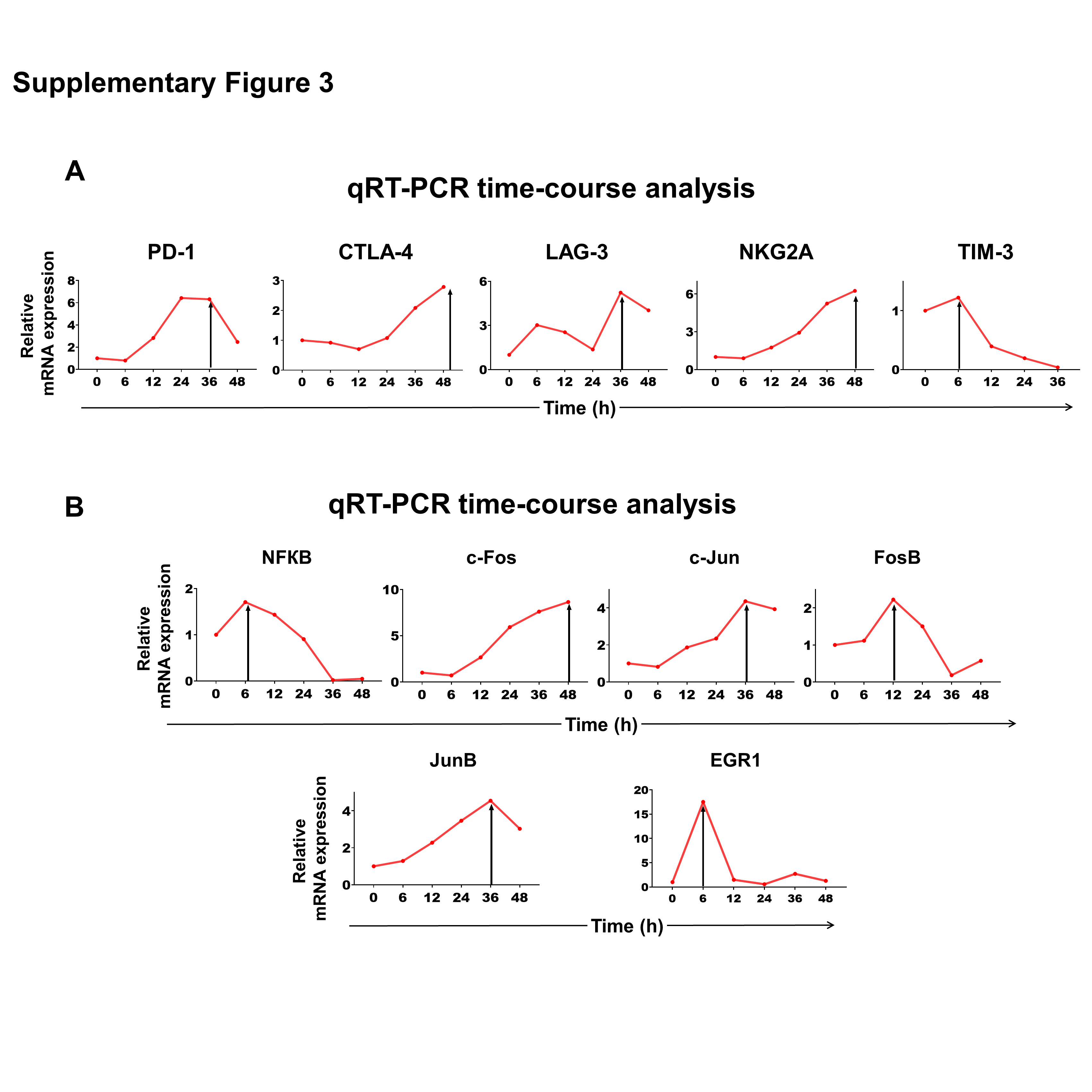

Supplement: Supplementary Figure 3 — Time-course analysis using qRT-PCR to determine the point of maximum expression of immune checkpoints and stress markers following treatment of PBMCs with conditioned media containing cfChPs released from hypoxia- induced dying HeLa cells. (A) Upregulation of immune checkpoints and (B) upregulation stress related markers. [file Image_3.tif]

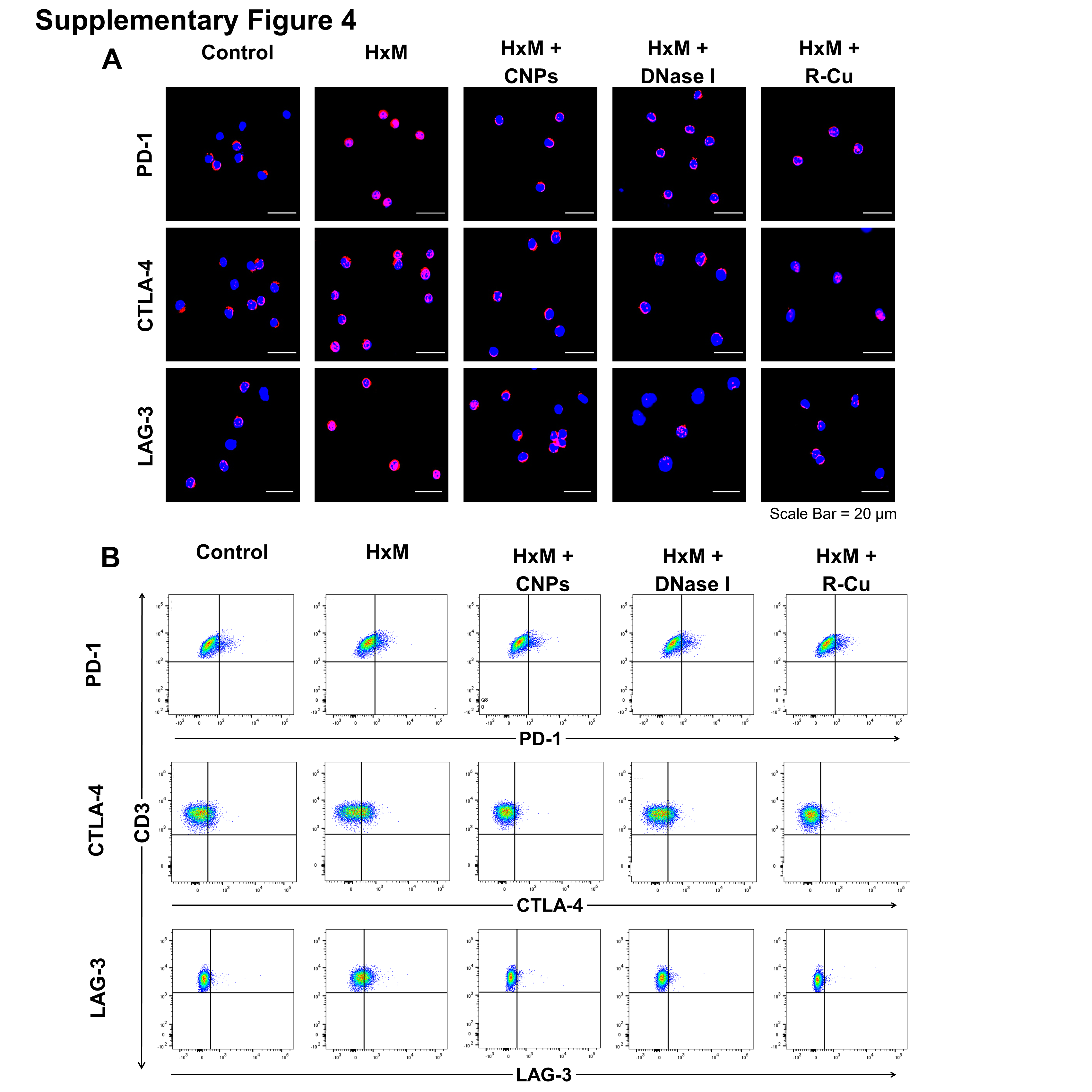

Supplement: Supplementary Figure 4 — Abrogation of immune checkpoints by cfChPs deactivators (A) Representative IF images showing immune checkpoint expression in T cells following treatment of PBMCs with conditioned media containing cfChPs released from hypoxia-induced dying HeLa cells. Pre-treatment of conditioned medium with CNPs (25μg), DNase I (0.005U) and R-Cu (1:10-4) significantly reduced the expression of immune checkpoints. (B) Representative flow cytometry plots showing immune checkpoint expression in T cells following treatment of PBMCs with conditioned media containing cfChPs released from hypoxia-induced dying HeLa cells. Pre-treatment of conditioned medium with CNPs (25μg), DNase I (0.005U) and R-Cu (1:10-4) significantly reduced the expression of immune checkpoints. [file Image_4.tif]
