# Supplementary material for: Cell-free chromatin particles released from dying cancer cells activate immune checkpoints in human lymphocytes: implications for cancer therapy
Source: Front Immunol. 2024 Jan 11;14:1331491. doi: 10.3389/fimmu.2023.1331491 (PMC10808321; doi:10.3389/fimmu.2023.1331491)
Supplement: Supplementary file 5 [file Table_1.docx]

| **Immune Checkpoint** | **Time of detection** | | | | | |
| --- | --- | --- | --- | --- | --- | --- |
|  | **CD4+ cells** | | | **CD8+ cells** | | |
|  | **qRT-PCR** | **IF** | **Flow cytometry** | **qRT-PCR** | **IF** | **Flow cytometry** |
| PD-1 | 12h | 12h | 72h | 6h | 6h | 72h |
| CTLA-4 | 12h | 12h | 72h | 6h | 6h | 72h |
| NKG2A | 6h | 6h | 72h | 6h | 6h | 72h |
| LAG-3 | 12h | 12h | 72h | 12h | 12h | 72h |
| TIM-3 | 36h | 36h | - | 48h | 48h | - |

**Supplementary Table 1:** Time-point of detecting immune checkpoint expression
